# Physicians’ acceptance of large language model–based clinical decision support tools in gynecologic oncology: a technology acceptance model study

**DOI:** 10.3389/fdgth.2026.1896184

**Published:** 2026-07-14

**Authors:** Jan Lennart Stalp, Juliane Alexandra Schneider, Anna Krause, Lena Steinkasserer, Jens Hachenberg, Agnieszka Denecke, Peter Hillemanns, Dominik Wolff

**Affiliations:** 1 Department of Obstetrics and Gynecology, Hannover Medical School, Hannover, Germany; 2 Commission Digital Medicine, German Society for Gynecology and Obstetrics (DGGG), Berlin, Germany; 3 Peter L. Reichertz Institute for Medical Informatics of TU Braunschweig and Hannover Medical School (PLRI), Hannover Medical School, Hannover, Germany

**Keywords:** clinical decision support system, large language model, medical guideline, survey, technology acceptance model

## Abstract

**Introduction:**

Medical decision-making is characterized by rapidly evolving evidence being represented by national and international guidelines that form the decision basis for treatment recommendations. Large language model-based clinical decision support systems (LLM-CDSS) have shown promising potential to serve as a support tool, e.g., in gynecologic oncology. Nevertheless, physicians’ acceptance of this technology in gynecologic oncology is currently not backed by evidence. Therefore, we present a pilot study evaluating the clinical technology acceptance of LLM-CDSS in gynecologic oncology.

**Methods:**

We designed a questionnaire based on the existing technology acceptance model (TAM) and the unified theory of acceptance and use of technology (UTAUT) characteristics, which was sent to clinicians employed at a clinic of gynecology and obstetrics of a large tertiary hospital. A total of 29 physicians spanning the complete range of clinical experience answered the survey. For identifying factors influencing the willingness to use a LLM-CDSS, Ordinary Least Squares (OLS) regression was applied based on answers grouped by question categories. Further, descriptive analysis was performed by measures of central tendency and correlation.

**Results:**

OLS regression did not identify any question categories significantly influencing willingness to use LLM-CDSS. Answer distribution was left skewed for the primary endpoint, indicating an acquiescence bias in the survey population. Still, descriptive findings indicate a generally positive but cautious attitude toward LLM-based therapy recommendation systems in gynecologic oncology. Acceptance appeared to depend strongly on transparency, integration of evidence, clinical validation, and the preservation of physician oversight and accountability.

**Discussion:**

The participants agreed on a possible positive influence of LLM-CDSS for clinical practice. However, the results emphasize that such tools should support rather than replace physicians to leave the final medical decision to the human. Participants were divided on whether patients must be informed about the usage of such tools whereas some participants reported concerns about their clinical autonomy and patients’ trust. The survey needs to be validated in a larger multicentered cohort in a shortened version.

## 1 Introduction

Large language models (LLMs) recently demonstrated substantial potential as clinical decision support tools, particularly in domains characterized by rapidly evolving evidence, complex treatment pathways, and multidisciplinary decision-making (1). Gynecologic oncology represents such a domain, where therapy recommendations are guided by international and national guidelines, molecular tumor characteristics, and tumor board deliberations. LLM-based systems that synthesize patient-specific information with up-to-date evidence and guideline recommendations could support clinicians in therapy planning, improve consistency of care, and reduce cognitive and time-related burdens (2). Existing quality-related limitations of such systems, especially in the field of breast cancer care, were identified for chemotherapy recommendations and complex/metastatic cases (3, 4). Further projects revealed promising recommendations for adjuvant treatment in early-stage breast cancer, considering medical preconditions (5). Regarding ovarian cancer therapy, a recent study by Cai et al. identified general inaccuracies and overly technical responses as significant limitations to its routine application (6). However, there is a lack of insight into the quality of decision support for cervical cancer cases. Recently developed preassessment LLMs have also shown potential for consultation support by synthesizing patient records, generating case summaries, preliminary diagnoses, and test ordering (7). While these findings highlight the promise of LLM-assisted clinical decision support, most authors emphasize the continued need for expert oversight, transparent evidence attribution, and prospective clinical validation before routine implementation (3–6). Despite growing technical feasibility, the successful implementation of LLM-based clinical decision support systems (LLM-CDSS) critically depends on physicians’ acceptance and intention to use such tools in routine practice. Concerns might relate to impaired trust, transparency, and data protection particularly in oncology and may influence adoption of decisions. Empirical evidence on physicians’ technology acceptance of LLM-based therapy recommendation tools in gynecologic oncology is currently lacking.

In 1989, Davis et al. introduced the technology acceptance model (TAM) as an information systems theory to mimic the user's way to approve and use a new technology. Key items are the *perceived usefulness* and the *perceived ease of use* that influence the actual system use (8). The model has been frequently applied in the past (9) and was expanded by Venkatesh et al., who evaluated eight existing models and incorporated them into the unified theory of acceptance and use of technology (UTAUT) in 2003. This model includes four key topics being *performance expectancy*, *effort expectancy*, *social influence*, and *facilitating conditions* to determine the usage intention (10). The TAM has recently been deployed in surveys about LLM acceptance among older adults (mean age 55 years) and for incorporating LLMs in medical studies among students both indicating that the perceived usefulness directly influenced the intention to use (11, 12). To our knowledge, neither the TAM nor the UTAUT has been used in the context of gynecologic oncology.

To evaluate the clinical technology acceptance of LLM decision support tools in gynecologic oncology, we designed a questionnaire based on the existing TAM and the UTAUT characteristics, with extensions regarding for instance *trust*, *expected risks* and *data safety*. Our pilot study will provide insight into the opinions of physicians at a German university hospital regarding LLM-based therapy recommendation tools in gynecological oncology and a survey design for future projects. It will also help identify obstacles to implementing these tools into daily clinical routines.

## 2 Material and methods

### 2.1 Design of survey

The survey was grounded in the TAM and its extensions, complemented by constructs from the UTAUT and recent literature on trust in artificial intelligence (AI) in healthcare (8, 10, 13, 14). Core TAM constructs—perceived usefulness and perceived ease of use—were integrated with additional factors particularly relevant to clinical AI applications. The questionnaire was designed as a survey to identify key points directly influencing clinicians’ intention to use LLM-CDSS. The survey started with a brief use-case scenario to guarantee a common knowledge basis, and an introductory presentation was given within a clinical meeting. Question categories in use were *expectations and needs*, *trust in AI-generated recommendations*, *clinical and social risks*, *social influence*, *data safety and ethics*, *perceived usefulness*, *intention to use*, *expected functionality*, *limitations*, two *clinical scenarios*, and *physicians’ subjective AI literacy*. Behavioral intention to use LLM-CDSS served as the primary outcome variable. Additionally, the questionnaire included demographic characteristics such as age, gender, clinical role, clinical experience, clinical specialty (e.g., obstetrics or gynecologic oncology), and overall context independent use of AI/LLM tools. All categories consisted of several questions with answer options based on a seven-point Likert scale (1 = no consent; 4 = indifferent; 7 = full consent). The survey was provided via the online platform *LimeSurvey* (LimeSurvey GmbH, Hamburg, Germany) using a campus license and is depicted in whole in Supplementary File 1.

### 2.2 Participating cohort

Participating clinicians were employed at the Department of Obstetrics and Gynecology at the Hannover Medical School (university hospital). In total, 38 physicians working in the clinic on a daily basis received the questionnaire. This included all levels of clinical training from the beginning of residency up to the head/chairman of the department. The hospital ranges on 5th place among German hospitals according to Newsweek World’s Best Hospitals (15) and the department lists in the ten biggest gynecologic oncology centers nationwide and treats all gynecologic malignancies regularly. The oncologic infrastructure additionally includes a center for breast cancer and a chemotherapy outpatient clinic. Therefore, all necessary framework conditions were given to serve as a representative single-center cohort.

### 2.3 Data analysis

Question ID is composed by group number (“G”) and number of the question within the group (“Q”), e.g., G1Q3. For the primary outcome (intended use, G7Q2), the median, inter-quartile range, and unbiased Fisher–Pearson standardized moment skewness are reported. For identifying influencing coefficients, data was mean grouped by question categories, an intercept added, and an Ordinary Least Squares (OLS) regression was performed on the grouped data. Data grouping was necessary due to the question-to-participant-proportion (41 questions to 29 participants) (16). During grouping, G11F6 (knowledge of the university's guidelines for working with AI) was excluded due to its binary scale. Due to left skewed data distribution of the primary variable and small sample size, heteroscedasticity robust covariance type 3 was applied for OLS. No further data transformation was applied to counter the skewness. A significance level of 5% was used. Further, correlation analysis on question group level and single question level was performed using the spearman rank correlation coefficient *ρ*. All analyses were performed in Python 3.10 using pandas (v1.3.3) (17, 18), seaborn (v0.13.2) (19), SciPy (v1.15.3) (20), Matplotlib (v3.10.8) (21, 22) and statsmodels (v0.14.6) (23).

## 3 Results

The participation rate was 76.32% (29 out of 38). The cohorts median age was 33 years with a median of 6 years of experience and 1.8 times more female than male participants. All clinical functionality levels were represented with assistant physicians and senior physicians as largest groups. Subjects’ clinical focus was mostly on obstetrics and prenatal medicine or gynecologic oncology. 20 of the 29 subjects were regular AI-Tool users (at least once a week) and almost two thirds attended the introductory presentation (see Table 1).

**Table 1:** Overview of the cohort’s demographics.

Demographic variable	Manifestation (*n* = 29)
Age	26–63 years median: 33.5 years 1 N/A
Gender	10 male 18 female 1 N/A
Functionality	13 assistant physician 4 specialist physician 8 senior physician 3 division head/chief physician
Professional experience	1–38 years median: 6 years
Clinical focus	15 none 5 Obstetrics/Prenatal Medicine 7 Gynecologic Oncology 1 Endocrinology and reproduction medicine 1 N/A
AI Usage	median: 4 IQR: 2
Attendance at presentation	19 yes 10 no

**Figure 1:**
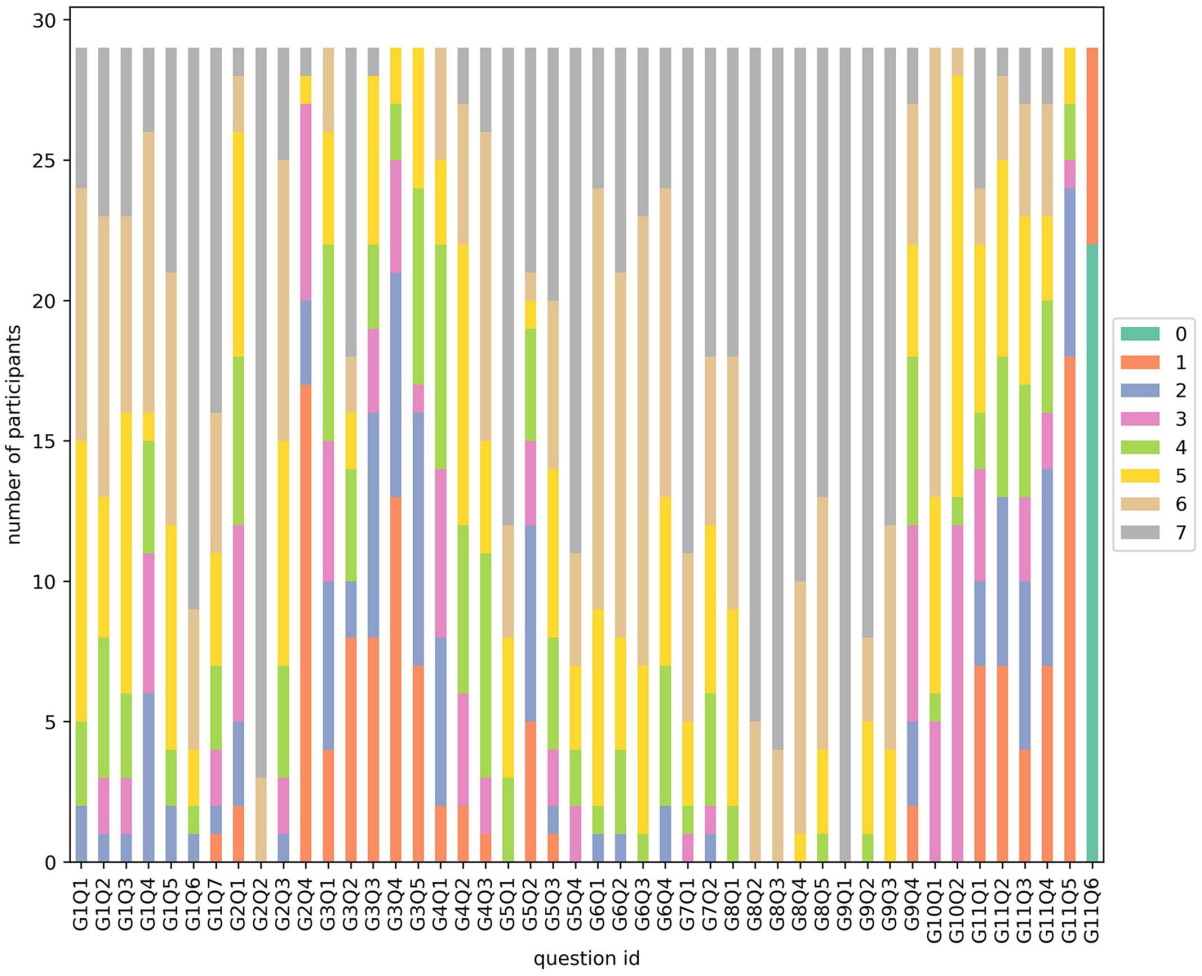
Distribution of the participants’ answers per question (*n* = 29 participants). The answer options for the survey questions were on a seven-item-Likert-scale, indicated as values 1−7 in the figure legend, except for G11Q6 which had binary answer options, indicated as values 0 (answer option “no”) and 1 (answer option “yes”).

**Figure 2:**
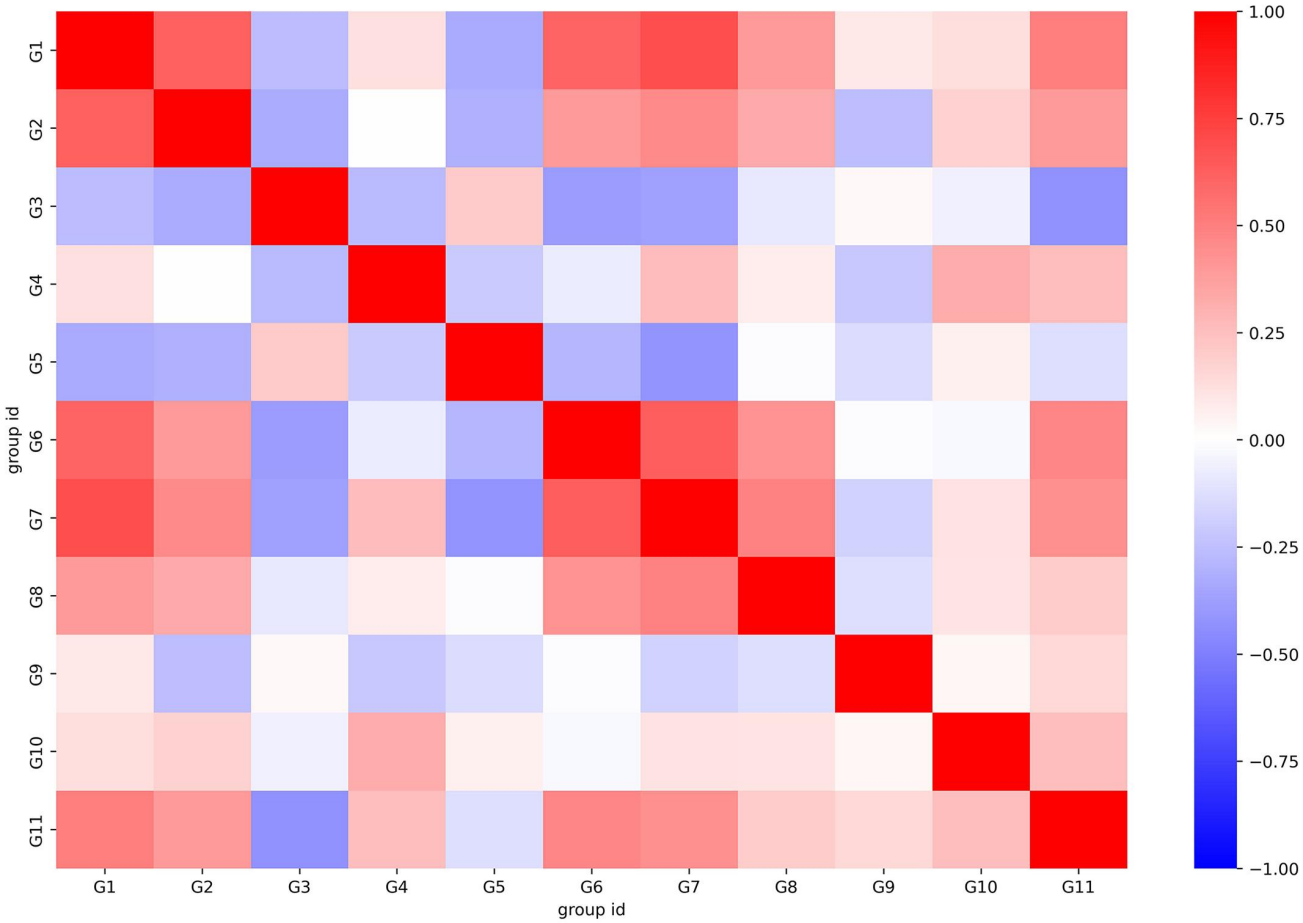
Pairwise correlation of the mean Likert-scale scores per question group using Spearman’s rank correlation coefficient *ρ*.

**Figure 3:**
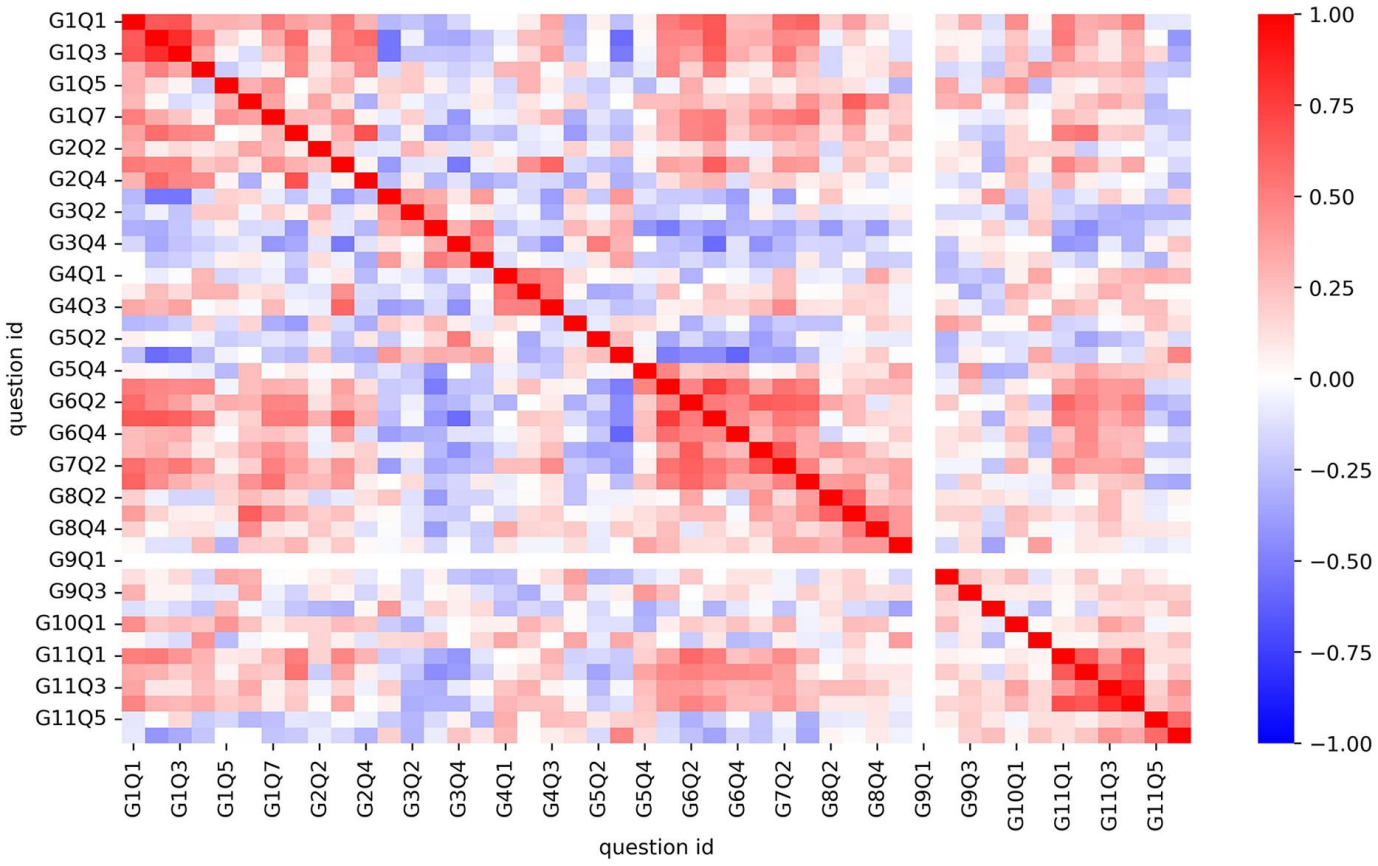
Pairwise correlation of the mean Likert-scale scores per question using Spearman’s rank correlation coefficient *ρ*.

Figure 1 shows an overview of the subjects’ answers. The primary outcome (intended use, question G7Q2) exhibits a median of 6 with an inter-quartile range of 2. The variable is left-skewed (−0.851). OLS did not identify any statistically significant variables associated with intended use (Table 2). Groups with the highest positive correlation to the primary endpoint group (G7) were *expectations and needs* (*ρ* = 0.69, G1) and *perceived usefulness* (*ρ* = 0.62, G6) while *data safety and ethics* (*ρ* = −0.41, G5) and *clinical and social risks* (*ρ* = −0.36, G3) were negatively correlated with the primary endpoint (see Figure 2). On single question level, willingness to try such a tool (*ρ* = 0.658, G7Q1), hopes for efficiency improvement (*ρ* = 0.6188, G6Q2), time savings for therapy planning (*ρ* = 0.5666, G1Q1), and improved clinical decision quality (*ρ* = 0.551, G6Q1) together with support in consistent implementation of evidence-based decisions (*ρ* = 0.5322, G6Q3) and hopes for more standardized tumor board decisions (*ρ* = 0.5244, G1Q3) showed moderate positive correlation with the primary outcome while fear of increased risk of medical errors (*ρ* = −0.3855, G3Q1), positive attitude of the department head towards LLM-CDSS (*ρ* = −0.3787, G4Q3) and concerns about clinical autonomy (*ρ* = −0.341, G3Q3) were weakly negatively correlated with the primary outcome (see Figure 3).

**Table 2:** Results of the ordinary least squares regression for mean Likert scale scores in question groups G1–G11.

Predictor/intercept	coef	std err	z	P>|z|	0.025	0.975
const	−8.3245	8.996	−0.925	0.355	−25.956	9.307
G1 (expectations and need)	−0.1552	0.538	−0.288	0.773	−1.210	0.900
G2 (Trust in AI-generated recommendations)	0.3662	0.542	0.676	0.499	−0.696	1.428
G3 (Risks)	0.0484	0.294	0.165	0.869	−0.528	0.625
G4 (Social influence)	0.4038	0.321	1.257	0.209	−0.226	1.033
G5 (Data safety and ethics)	0.1181	0.378	0.313	0.754	−0.622	0.858
G6 (Perceived usefulness)	0.8725	0.666	1.309	0.190	−0.433	2.178
G7 (Intention to use)	0.4264	0.345	1.235	0.217	−0.250	1.103
G8 (Expected functionality)	0.3663	0.682	0.537	0.591	−0.970	1.702
G9 (Limitations)	0.0182	0.649	0.028	0.978	−1.255	1.291
G10 (Clinical scenarios)	0.1510	0.290	0.521	0.602	−0.417	0.719
G11 (AI literacy)	−0.0803	0.239	−0.336	0.737	−0.549	0.389

Overall, respondents demonstrated a favorable attitude toward the implementation of LLM-CDSS in gynecologic oncology. The strongest agreement was observed for the statement that such systems should serve exclusively as decision-support tools while the final clinical decision remains with the physician (median = 7, iqr = 0, G9Q1). Similarly, participants strongly agreed that uncertainty generated by the system should be communicated transparently (median = 7, iqr = 0, G2Q2), and that patient-specific clinical factors should be incorporated into the recommendation process (median = 7, iqr = 0, G8Q3). Respondents also emphasized the importance of integrating current guidelines and scientific evidence into the system (median = 7, iqr = 0, G8Q2), as well as providing evidence levels and literature references alongside recommendations (median = 7, iqr = 1, G8Q4).

High agreement was additionally observed regarding safety- and governance-related requirements. Participants strongly supported mandatory human co-signature of recommendations (median = 7, iqr = 1, G9Q2) and the prerequisite of indication-specific clinical validation before implementation (median = 7, iqr = 1 G9Q3). The majority of respondents agreed that LLM-based tools should support rather than replace clinical reasoning processes (median = 7, iqr = 1, G1Q6).

The perceived usefulness of the technology was generally high. Respondents indicated that LLM-based systems could improve their quality of clinical decisions (median = 6, iqr = 1, G6Q1), increase efficiency in therapy planning (median = 6, iqr = 2, G6Q2), support more consistent evidence-based decision-making (median = 6, iqr = 0, G6Q3), and potentially improve patient safety (median = 6, iqr = 1, G6Q4). In addition, there was a clear intention to test such systems in clinical practice (median = 7, iqr = 1, G7Q1), whereas willingness to use them regularly in routine therapy planning was slightly lower but remained positive overall (median = 6, iqr = 2, G7Q2).

The survey also revealed a substantial perceived need for digital decision-support solutions. Respondents expected time savings in therapy planning (median = 5, iqr = 1 G1Q1), improved standardization of tumor board recommendations (median = 5, iqr = 1, G1Q3), and a reduction in errors or guideline deviations (median = 6, iqr = 2, G1Q2). Support for routine clinical cases was rated more favorably (median = 6, iqr = 2, G1Q5) than support for complex cases (median = 5, iqr = 3, G1Q4).

Trust-related responses demonstrated a differentiated pattern. While respondents considered the system potentially reliable in standard clinical situations (median = 5, iqr = 1 G2Q3), general trust in system-generated recommendations was moderate (median = 4, iqr = 2 G2Q1). Notably, physicians strongly rejected the idea of adopting recommendations without prior verification (median = 1, iqr = 2, G2Q4), underscoring the continued importance of human oversight and critical appraisal.

The risks perceived associated with LLM-based decision support were moderate overall. Concerns regarding an increased risk of incorrect clinical decisions achieved intermediate agreement levels (median = 3, iqr = 2, G3Q1). Similarly, uncertainty regarding legal accountability remained relevant among respondents (median = 5, iqr = 6, G3Q2). In contrast, concerns about negative effects on the physician–patient relationship (median = 2, iqr = 2, G3Q4), negative patient perceptions (median = 2, iqr = 2, G3Q5), or reduced clinical autonomy (median = 2, iqr = 3, G3Q3) were comparatively low.

Social influence factors were less pronounced than perceived usefulness or governance-related aspects. Expectations from supervisors and colleagues regarding the use of such systems received relatively low agreement (median = 4, iqr = 2, G4Q1), whereas support by the multidisciplinary tumor boards was rated somewhat higher (median = 5, iqr = 1, G4Q2).

Finally, self-reported AI literacy and prior experience with LLM technologies varied considerably among participants. While practical experience with LLMs in clinical contexts was moderate (median = 4, iqr = 3, G11Q1), self-assessed competence in using LLM-based tools was comparatively lower (median = 3, iqr = 3 G11Q4). Only a minority (24%) of respondents reported familiarity with existing AI-related clinical guidelines or recommendations at Hannover Medical School.

Taken together, the descriptive findings indicate a generally positive but cautious attitude toward LLM-based therapy recommendation systems in gynecologic oncology. Acceptance was associated with high-rated requirements such as transparency, evidence integration, clinical validation, and the preservation of physician oversight and accountability.

## 4 Discussion

Overall, the participants showed high willingness to use an LLM-CDSS for therapy planning in gynecologic oncology. The usage scenario in favor is distinct: a tool should support clinical decision making without replacing physicians. A final medical review of the decision was highly agreed upon by the participants, meaning that the final decision remains with the treating physician. Still, participants were ambiguous whether patients should be informed of the usage of an LLM-CDSS. Some participants saw risks in reduced patient's confidence in their abilities or their clinical autonomy. On the other hand, they agreed that LLM-CDSS would positively influence treatment and healthcare but must comply with the General Data Protection Regulation. Participants reported logging of prompts and system's outputs as well as clearly stating uncertainty if applicable as quality control mechanisms of high importance, which coincides with Regulation (EU) 2024/1689 (Artificial Intelligence Act) Chapter 3 Article 12 and Chapter 3 Articles 13 and 15 respectively (24). The observed acceptance pattern likely reflects clinicians’ pragmatic risk–benefit assessment: LLM-based tools are perceived as valuable for reducing cognitive workload, improving access to guideline-based knowledge, and supporting standardization, but only if their outputs remain transparent, verifiable, and subordinate to physician judgment. Importantly, answer quality is not an intrinsic property of the base model alone, but can be improved through careful prompt engineering, retrieval-augmented generation (RAG), and iterative clinical validation (25). RAG can anchor model responses in curated sources such as guidelines, institutional protocols, and peer-reviewed literature, thereby improving factual consistency and reducing unsupported claims (26). Nevertheless, hallucinations remain a central safety concern. In clinical contexts, hallucinations may include fabricated references, incorrect guideline interpretation, omitted contraindications, or plausible but unsafe therapeutic suggestions (27). Therefore, it is essential that the system discloses uncertainties, offers an audit trail, and cites the retrieved evidence. This is in line with existing studies that determined perceived benefits, performance and trust as strong predictors of intention to adopt AI based CDSS into healthcare in general (28, 29). Nevertheless, insight and verification for the field of gynecologic oncology was so far missing.

Regarding the primary outcome variable, answer distribution was left skewed which could be an indicator for an acquiescence bias in the survey population (30, 31). We addressed technology advantages as well as risks in the survey to minimize the risk of one-sided framing and the associated guidance bias. Although the survey was conducted anonymously, the observed skewness could also be a sign of induction bias. This bias might be due to a generally positive attitude toward technology among the group of respondents, potentially influenced by a currently overall positive attitude of society towards these new technologies. Therefore, the observed skewness could also be a sign of induction bias. Since the mindset of the corresponding healthcare professional and their technology confidence directly affects the perceived usefulness (32), the cohort selection plays a crucial role for representative results. Another possible explanation is the single center design of the survey. All participants are from a large tertiary care hospital that is also a university clinic. Therefore, participants are not necessarily representative members of the broad types of healthcare providers. Here, the subgroups of private practitioners, regional hospitals and centers without a gynecologic oncology focus were not represented. Therefore, our results cannot be generalized to those facilities, and further investigation is needed. Nevertheless, the cohort encompasses the entire spectrum of professional qualifications, ranging from resident physicians to clinic management to represent a university department.

The OLS regression did not reveal any significant influencing factors, potentially due to the grouping of questions. In this context, the significance of individual factors may be overshadowed by the other questions in the same group. For a regression analysis on single item level, the ratio of questions to participants was high (16) and even on group level the ratio is limited. Typically, a ten-to-one-ratio of participants to items is proposed (33). However, correlation analysis on single question level revealed potential influences. Our results are in line with technology acceptance studies from other medical domains, where it was shown that the perceived usefulness is a main influencing factor (11, 12). The survey presented here is to our knowledge the first technology acceptance model study for LLM-CDSS in gynecologic oncology. Our work serves as the basis for developing and validating a standardized questionnaire. For validation, the questionnaire can be shortened and validated in a larger multicentered setting. Questions that strongly correlated with one another (*ρ* > 0.7) may be removed for further studies. In our survey, highly correlated questions were G1Q2 and G1Q3, G6Q1 and G6Q3, as well as G11Q4 with G11Q1, G11Q2 and G11Q3 most likely due to linguistic variations that were irrelevant to the meaning. Hence, we provided an improved version for further studies within Supplementary File 2.

## 5 Conclusion

We presented the results of—to our knowledge—the first technology acceptance study on large language model-based clinical decision support systems in gynecologic oncology. Our cohort indicated high intention to use such systems if they aimed to support and not replace physicians. Based on the results of this study, a standardized validation of a reduced version of the survey in a larger multicenter cohort is intended.

## Data Availability

The original contributions presented in the study are included in the article/Supplementary Material, further inquiries can be directed to the corresponding authors.

## Author's note

DeepL (34) was used for wording of the manuscript itself and translation of the questionnaire. After using this service, the authors reviewed and edited the content as needed and take full responsibility for the content of the publication.
