# Supplementary material for: Physicians’ acceptance of large language model–based clinical decision support tools in gynecologic oncology: a technology acceptance model study
Source: Front Digit Health. 2026 Jul 14;8:1896184. doi: 10.3389/fdgth.2026.1896184 (PMC13410770; doi:10.3389/fdgth.2026.1896184)
Supplement: Supplementary file 2 [file Datasheet2.pdf]

Modified survey: Technology Acceptance of LLM-Based Treatment Recommendation Tools in Gynecologic Oncology

This survey is addressed to physicians working in the field of gynecology and obstetrics. Please answer the following questions. All responses are anonymous and will be treated confidentially.

Introduction

Consent: By participating in this anonymous survey, you consent to the processing of your information for research purposes. Your information will be treated confidentially and processed in accordance with the DSGVO (Datenschutz-Grundverordnung = General Data Protection Regulation). Participation is voluntary; you may withdraw at any time.

1. I have read the information and agree to participate.
   Answer options: 1=Yes; 0=No

00 Clinical vignette

During the morning training session on November 11, 2025, you were introduced to an LLM-based tool (similar to a large language model trained on current medical guidelines) that suggests treatment options for gynecological tumors based on patient records, tumor board protocols, and current guidelines (e.g., AGO). If you were unable to attend the morning training session, please envision such a LLM-based tool. To use the tool, you simply need to provide it with the structured tumor board minutes and enter a predefined query. The tool then provides a reasoned recommendation, including references, documents uncertainties, and logs entries locally at the hospital. The generated recommendation should be reviewed by a specialized body and ultimately approved by the tumor board.

01 *Expectations and needs*

1. I expect an LLM tool to save me time in treatment planning.
   Response options: 1=strongly disagree; 2; 3; 4=neutral; 5; 6; 7=strongly agree

2. I expect an LLM tool to reduce errors and deviations from guidelines.
   Response options: 1=strongly disagree; 2; 3; 4=neutral; 5; 6; 7=strongly agree

3. I would like an LLM tool to provide support, particularly in complex cases with multiple lines of therapy.
   Response options: 1=strongly disagree; 2; 3; 4=neutral; 5; 6; 7=strongly agree

4. I would like an LLM tool to provide support, especially for mild cases, so that I can focus on the more severe ones.
   Response options: 1=strongly disagree; 2; 3; 4=neutral; 5; 6; 7=strongly agree

5. It is important to me that an LLM tool supports my own clinical decision-making process, but does not replace it.
   Response options: 1=strongly disagree; 2; 3; 4=neutral; 5; 6; 7=strongly agree

6. I generally see a need for digital decision-making tools in gynecologic oncology.

Response options: 1=strongly disagree; 2; 3; 4=neutral; 5; 6; 7=strongly agree

02 Trust in AI-generated recommendations

1. I would generally trust the tool's recommendations.

Response options: 1=strongly disagree; 2; 3; 4=neutral; 5; 6; 7=strongly agree

2. If the tool is uncertain about the treatment recommendation from a technical standpoint, it should communicate this transparently (e.g., percentage values, confidence scores).

Response options: 1=strongly disagree; 2; 3; 4=neutral; 5; 6; 7=strongly agree

3. I consider such a tool to be reliable in common standard situations.

Response options: 1=strongly disagree; 2; 3; 4=neutral; 5; 6; 7=strongly agree

4. I would adopt the tool's recommendations without further review.

Response options: 1=strongly disagree; 2; 3; 4=neutral; 5; 6; 7=strongly agree

03 Risks

1. I see an increased risk of medical errors resulting from the use of such a tool.

Response options: 1=strongly disagree; 2; 3; 4=neutral; 5; 6; 7=strongly agree

2. I am unsure who is liable in the event of errors (me, the department, the hospital, the manufacturer).

Response options: 1=strongly disagree; 2; 3; 4=neutral; 5; 6; 7=strongly agree

3. I am concerned that using this tool could compromise my clinical autonomy.

Response options: 1=strongly disagree; 2; 3; 4=neutral; 5; 6; 7=strongly agree

4. I am concerned that its use could negatively impact the doctor-patient relationship.

Response options: 1=strongly disagree; 2; 3; 4=neutral; 5; 6; 7=strongly agree

5. I am concerned that its use could diminish patients' assessment of medical competence.

Response options: 1=strongly disagree; 2; 3; 4=neutral; 5; 6; 7=strongly agree

04 Social influence

How do you assess the level of acceptance in your work environment?

1. My supervisors and colleagues would expect me to use such a tool.

Response options: 1=strongly disagree; 2; 3; 4=neutral; 5; 6; 7=strongly agree

2. My tumor board would support the use of such a tool.

Response options: 1=strongly disagree; 2; 3; 4=neutral; 5; 6; 7=strongly agree

3. The department heads at my institution would endorse the use of such a tool.

Response options: 1=strongly disagree; 2; 3; 4=neutral; 5; 6; 7=strongly agree

05 Data safety and ethics

1. Use is acceptable only if all data is processed in compliance with the GDPR.

Response options: 1=strongly disagree; 2; 3; 4=neutral; 5; 6; 7=strongly agree

2. Use is acceptable only if patients give their informed consent.

Response options: 1=strongly disagree; 2; 3; 4=neutral; 5; 6; 7=strongly agree

3. I consider comprehensive documentation of the decision-making process to be necessary.

Response options: 1=strongly disagree; 2; 3; 4=neutral; 5; 6; 7=strongly agree

4. Prospective clinical validation (e.g., studies/CE) is a prerequisite for potential use.
Response options: 1=strongly disagree; 2; 3; 4=neutral; 5; 6; 7=strongly agree

06 Perceived usefulness

1. Such a tool could increase my efficiency in treatment planning.
Response options: 1=strongly disagree; 2; 3; 4=neutral; 5; 6; 7=strongly agree

2. Such a tool could help implement evidence-based decisions more consistently.
Response options: 1=strongly disagree; 2; 3; 4=neutral; 5; 6; 7=strongly agree

3. Such a tool could improve patient safety.
Response options: 1=strongly disagree; 2; 3; 4=neutral; 5; 6; 7=strongly agree

07 Intention to use

1. I would try out such a tool as soon as it becomes available.
Response options: 1=strongly disagree; 2; 3; 4=neutral; 5; 6; 7=strongly agree

2. I would use such a tool regularly in therapy planning.
Response options: 1=strongly disagree; 2; 3; 4=neutral; 5; 6; 7=strongly agree

08 Expected functionality

How important is it to you that…

1. …such a tool integrates seamlessly into existing systems.
Response options: 1=unimportant; 2; 3; 4=neutral; 5; 6; 7=essential

2. …such a tool automatically incorporates current guidelines and studies.
Response options: 1=unimportant; 2; 3; 4=neutral; 5; 6; 7=essential

3. …such a tool takes patient-specific factors (e.g., age, comorbidities, genetic markers) into account.
Response options: 1=unimportant; 2; 3; 4=neutral; 5; 6; 7=essential

4. … the recommendations are supported by evidence levels, source citations, and links to studies.
Response options: 1=unimportant; 2; 3; 4=neutral; 5; 6; 7=essential

5. …such a tool provides audit/log functions to document decisions in a traceable manner.
Response options: 1=unimportant; 2; 3; 4=neutral; 5; 6; 7=essential

09 Limitations

1. The tool should only be used as a decision-making aid; the final decision is made by the physician.
Response options: 1=strongly disagree; 2; 3; 4=neutral; 5; 6; 7=strongly agree

2. The tool should only be used with mandatory human approval (dual-control principle).
Response options: 1=strongly disagree; 2; 3; 4=neutral; 5; 6; 7=strongly agree

3. The tool should only be used after successful clinical validation for the respective indication.
Response options: 1=strongly disagree; 2; 3; 4=neutral; 5; 6; 7=strongly agree

4. The system should only be used for standard cases, not for complex exceptions.
Response options: 1=strongly disagree; 2; 3; 4=neutral; 5; 6; 7=strongly agree

10 Clinical scenarios

1. When making a first-line treatment decision (FIGO IIIC ovarian cancer), I would handle the tool's recommendation as follows...

Response options: 1=reject without reviewing; 2=review but do not use; 3=consider as an option after review; 4=don't know; 5use as a guide after review; 6=adopt after review; 7=adopt without review

2. If the guidelines are unclear, I would handle the tool recommendation...

Response options: 1=reject without review; 2=review but not use; 3=consider as an option after review; 4=don't know; 5=use as a guide after review; 6=adopt after review; 7=adopt without review

11 AI literacy

1. I have practical experience with LLMs (e.g., ChatGPT) in a clinical setting.

Response options: 1=strongly disagree; 2; 3; 4=neutral; 5; 6; 7=strongly agree

2. I am familiar with the current guidelines/position papers on the use of AI in medicine.

Response options: 1=strongly disagree; 2; 3; 4=neutral; 5; 6; 7=strongly agree

3. I am familiar with the Hannover Medical School (MHH) guidelines on the use of artificial intelligence (AI)

Response options: yes/no

12 Quality

1. Attention check: Please select "strongly agree" (7) here.

Response options: 1 = strongly disagree; 2; 3; 4 = neutral; 5; 6; 7 = strongly agree

13 Demographics

1. Your age:

Answer: Please enter a number

2. Gender:

Answer options: female; male; diverse; not specified

3. Position/role:

Answer options: Resident; Specialist; Senior Resident; Division Head/Clinical Chief

4. Total years of clinical experience:

Answer: Please enter a number

5. Specialty:

Answer options: gynecologic oncology; breast cancer care; reproductive medicine; obstetrics/prenatal medicine; currently not specialized

6. General current use of AI tools (including LLMs) (frequency):

Answer options: 1=never; 2=less than once a month; 3=once a month; 4=once a week; 5=several times a week; 6=daily; 7=several times a day

7. I attended the morning training session on November 10, 2025, led by L. Stalp and D. Wolff on the topic of AI tools in gynecologic oncology.

Answer options: Yes/No

14 Open Feedback

1. Open feedback / concerns (optional):

Response: Free text
